# dbDEMC 3.0: Functional Exploration of Differentially Expressed miRNAs in Cancers of Human and Model Organisms

**DOI:** 10.1016/j.gpb.2022.04.006

**Published:** 2022-05-25

**Authors:** Feng Xu, Yifan Wang, Yunchao Ling, Chenfen Zhou, Haizhou Wang, Andrew E. Teschendorff, Yi Zhao, Haitao Zhao, Yungang He, Guoqing Zhang, Zhen Yang

**Affiliations:** 1 Center for Medical Research and Innovation of Pudong Hospital, Fudan University Pudong Medical Center, Shanghai 201399, China; 2 Institutes of Biomedical Science, Shanghai Key Laboratory of Medical Epigenetics, International Co-laboratory of Medical Epigenetics and Metabolism (Ministry of Science and Technology), Fudan University, Shanghai 200032, China; 3 Bio-Med Big Data Center, CAS Key Laboratory of Computational Biology, Shanghai Institute of Nutrition and Health, University of Chinese Academy of Sciences, Chinese Academy of Sciences, Shanghai 200031, China; 4 CAS Key Laboratory of Computational Biology, Shanghai Institute of Nutrition and Health, University of Chinese Academy of Sciences, Chinese Academy of Sciences, Shanghai 200031, China; 5 Institute of Computing Technology, Chinese Academy of Sciences, Beijing 100190, China; 6 Department of Liver Surgery, Peking Union Medical College Hospital, Chinese Academy of Medical Sciences and Peking Union Medical College, Beijing 100730, China; 7 Shanghai Fifth People’s Hospital, Fudan University, Shanghai 200240, China

**Keywords:** MicroRNA, Cancer, Differential expression, Model organism, Database

## Abstract

**MicroRNAs** (miRNAs) are important regulators in gene expression. The dysregulation of miRNA expression is widely reported in the transformation from physiological to pathological states of cells. A large number of differentially expressed miRNAs (DEMs) have been identified in various human **cancers** by using high-throughput technologies, such as microarray and miRNA-seq. Through mining of published studies with high-throughput experiment information, the **database** of DEMs in human cancers (dbDEMC) was constructed with the aim of providing a systematic resource for the storage and query of the DEMs. Here we report an update of the dbDEMC to version 3.0, which contains two-fold more data entries than the second version and now includes also data from mice and rats. The dbDEMC 3.0 contains 3268 unique DEMs in 40 different cancer types. The current datasets for **differential expression** analysis have expanded to 9 generalized categories. Moreover, the current release integrates functional annotations of DEMs obtained by using experimentally validated targets. The annotations can be of great benefit to the intensive analysis of the roles of DEMs in cancer. In summary, dbDEMC 3.0 provides a valuable resource for characterizing molecular functions and regulatory mechanisms of DEMs in human cancers. The dbDEMC 3.0 is freely accessible at https://www.biosino.org/dbDEMC.

## Introduction

Since the first discovery of microRNAs (miRNAs) at the beginning of this century, this class of small non-coding RNAs has received extensive attention [1]. As an important gene expression regulator acting at the post-transcriptional level, studies have disclosed the critical role of miRNAs in targeting mRNAs for the degradation or translational repression [2]. A total of 2654 miRNAs have been identified in the human genome according to the latest version of miRBase database [3]. Vast researches on miRNAs have dramatically expanded our understanding about gene regulatory network and their roles in physiological and pathological conditions, such as in broad spectra of biological processes including cell cycle, cell proliferation, differentiation, apoptosis, and cellular signaling [4], [5]. Owing to the biological significance of miRNAs, alterations of their expression have been linked to the development of many diseases including the cancer [6]. Differentially expressed miRNAs (DEMs) are widely reported to hold great value in the diagnosis or prognosis as well as treatment targeting for cancer research [7]. The potential usage of circulating miRNAs in serum, plasma, and other body fluids as non-invasive cancer biomarkers has also been thoroughly investigated [8].

Given the important functions of miRNAs in cancer development, several on-line resources have been built for warehousing information of cancer-related miRNAs, such as the HMDD [9], miRCancer [10], and OncomiRDB [11]. With the development of high-throughput techniques such as microarray and miRNA-seq, large amount of cancer DEMs were identified from miRNA profiling data each year. However, these valuable data are scattered in the vast literature and it is of great necessity to catalogue them in a favourable way, thus to provide integrative tools for the effective utilization and systematic investigation. With this aim, we developed the initial database of DEMs in human cancers (dbDEMC) in 2010 [12] and further updated it in 2017 [13]. To our knowledge, dbDEMC is the only working repository currently available for storing DEMs from *de novo* analysis of high-throughput profiling data in human cancers, which is characteristic with miRNANome data in various types of cancer. It greatly facilitates the efforts to excavate cancer-associated miRNAs and investigate their roles in the pathological processes of cancer. While the database could have been much more useful if there been more high-quality data included.

In recent years, cancer quantitative miRNA profiling data have been increasing at an unprecedented rate, and given the success of dbDEMC 2.0, this motivates an update of this database. Here we introduce dbDEMC 3.0, a significantly expanded version of this database. This update incorporates a substantial amount of new data. Besides the human data, we have also incorporated the miRNA expression profiling data of mouse and rat. A total of 403 datasets of miRNA high-throughput expression encompassing 40 cancer types, with the results of 807 differential expression analyses, have been included. The present update is nearly doubling the data amount over the previous version. In addition to the expanded data volume, the content of the database has also been enriched. This new version incorporates the experimentally validated DEM targets and also their enrichment analysis results on Gene Ontology (GO) terms and Kyoto Encyclopedia of Genes and Genomes (KEGG) pathways for the first time. In this way, we provide the functional annotations of DEMs for various cancers. Also, the web interface of the database has been refined for a better visualization of the aforementioned data. Taken together, the dbDEMC 3.0 is a comprehensive resource to systematically characterize the function of DEMs in human cancers as well as other model organisms.

## Data collection and processing

### Data collection

To compile the datasets, we used the keywords “cancer”, “tumor”, “carcinoma”, and “neoplasm”, in combination with “microRNA” or “miRNA” to conduct an exhaustive search for the microarray-based miRNA expression profiles in Gene Expression Omnibus (GEO) [14] and ArrayExpress [15], and further for miRNA-seq-based miRNA expression profiles in Sequence Read Archive (SRA) [16]. While the miRNA profiles of mouse and rat were rapidly accumulating, we also incorporated the miRNA data of the two model organisms in the current update. In addition, we also appended the miRNA profiling data from The Cancer Genome Atlas (TCGA) that was newly released since the last update of dbDEMC 2.0. All the involved data were published before June 2021. The data records were manually reviewed and evaluated rigorously to guarantee that only high-quality datasets were included. To ensure analysis reliability, we required at least three biological replicates of samples in each condition (for both case and control) as usual.

### Data processing

For miRNA profiling datasets based on microarray, we used the same protocol as that of dbDEMC 2.0 to identify the DEMs [13]. Briefly, the expression values were logarithmically transformed (base 2) and quantile normalized. Then the limma (Linear Models for Microarray Data) package was applied to select miRNAs whose mean expression level was significantly different between case and control samples with false discovery rate (FDR) < 0.05.

For miRNA-seq-based profiling data obtained from SRA database, we downloaded the SRA files of raw sequence reads and converted them into FASTQ format using the fastq-dump of SRA Toolkit. Here we only used the data produced by Illumina systems (Genome Analyzer I, II, IIx, HiSeq 1000, HiSeq 2000, HiSeq 2500, HiSeq 4000, NextSeq, and MiSeq). The involving miRNA-seq data were analyzed by using QuickMIRSeq toolkit [17]. This toolkit utilizes the Cutadapt to remove sequence adapters and perform quality control [18]. We collected detailed information of DNA adapters of different miRNA-seq libraries from public resources to guarantee that the adapters can be properly trimmed from the raw reads (Table S1) [19]. The clean reads were then aligned to the reference genome by using Bowtie [20], and miRDeep2 was used to obtain count tables of aligned reads for miRNA quantification [21]. The read count table was further normalized by using limma-voom [22], and DEMs were then identified. For the datasets obtained from TCGA, we directly used the read count data provided by the their data portal for further analysis [23].

Experimental validation results of DEMs in low-throughput methods, such as real-time polymerase chain reaction (RT-PCR) and Northern blot, were manually collected from the original papers. These types of information were carefully formatted and integrated into our update.

### Functional annotation

**Figure 1:**
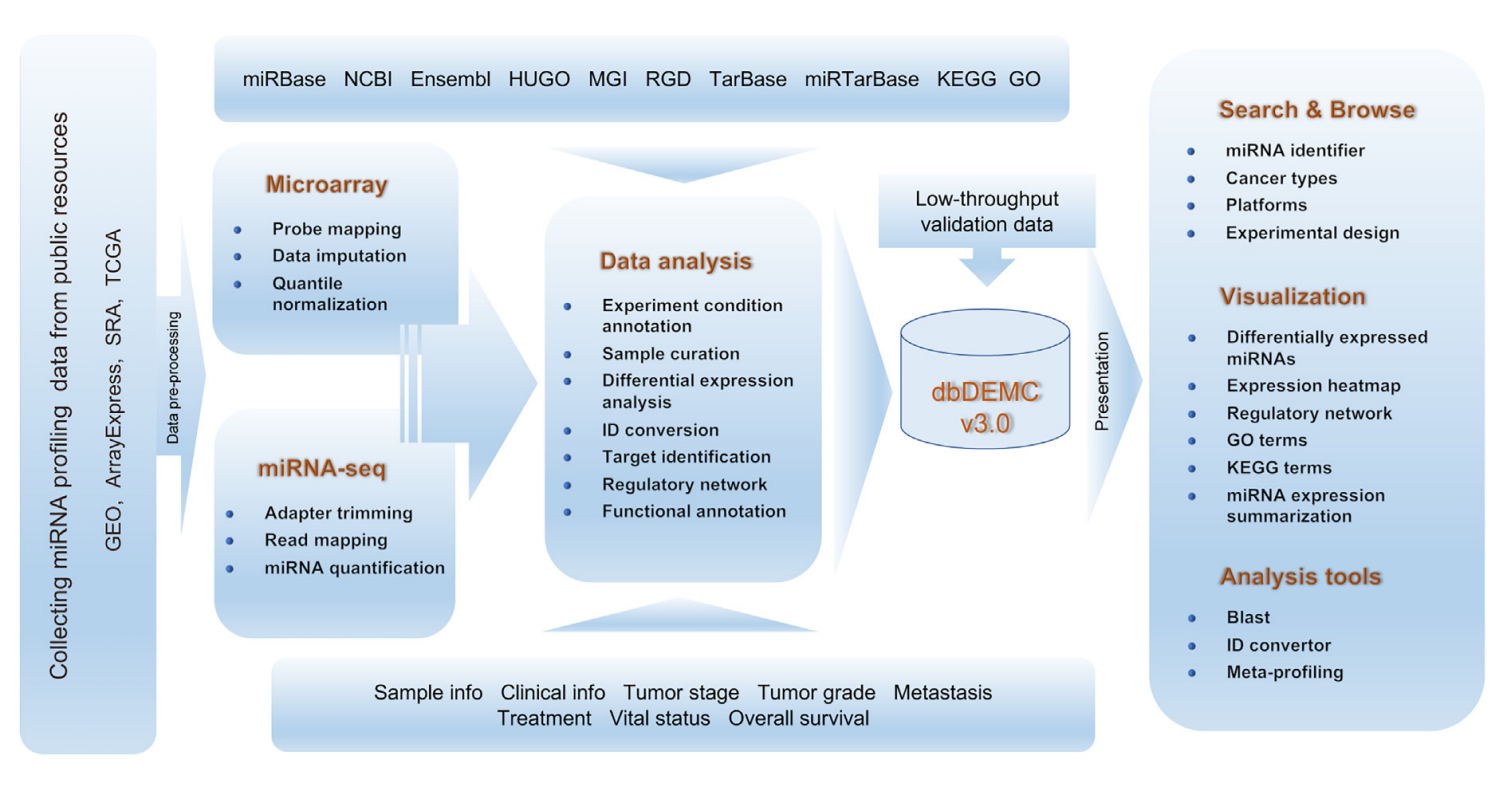
**Schematic illustration of the data collection and architecture of the dbDEMC 3.0** GEO, Gene Expression Omnibus; SRA, Sequence Read Archive; TCGA, The Cancer Genome Atlas; NCBI, National Center for Biotechnology Information; HUGO, Human Genome Organisation; MGI, Mouse Genome Informatics; RGD, Rat Genome Database; KEGG, Kyoto Encyclopedia of Genes and Genomes; GO, Gene Ontology.

For each obtained DEM set, we collected the experimentally validated targets by using multiMiR [24], which integrate miRNA target data from TarBase [25] and miRTarBase [26]. Then we performed the enrichment analysis of the DEM targets on GO terms and KEGG pathways by using clusterProfiler package to facilitate the study of context-dependent miRNA functional mechanisms [27]. Enriched GO terms and KEGG pathways were selected where adjusted *P* value < 0.05. The data collection and curation procedure for dbDEMC 3.0 is shown in Figure 1.

### Database construction

All the data in dbDEMC 3.0 were managed by using MongoDB. The dynamic web interface was developed using Java Server Pages (JSP) and JavaScript. Data visualization was achieved through the tools of vue, jQuery, and Echarts, and Elasticsearch was used for search engine. The database was developed by Spring Boot framework. Apache Tomcat was used for the http server. All the information in dbDEMC 3.0 is freely available to the public domain through https://www.biosino.org./dbDEMC.

### miRNA cluster annotation

A miRNA cluster is defined as a set of miRNAs which are located within adjacent genomic regions in the same or opposite orientation and not separated by other transcriptional units. miRNAs within a cluster are thought to be regulated by common factors and involved in same signaling pathways. According to Kabekkodu SP et al. [28], among 1881 precursor miRNAs of human origin annotated in miRBase, 468 can be attributed to 153 clusters. Here we obtained these data about miRNA clusters and annotated mature miRNAs by using annotation file from miRBase. Finally, a total of 688 (22.8%) mature miRNAs from 143 clusters were annotated in the human genome.

### Analysis of homogeneous dysregulation pattern of miRNA clusters in cancer

For the systematic study of co-dysregulation pattern of miRNA clusters in human cancers, we considered all miRNAs associated with specific cancer. miRNAs not belonging to any cluster and clusters of which at least half the members are not associated with any cancer were discarded. To avoid potential bias introduced by different expression platforms, here we only used the results obtained from TCGA and checked for experimental design of Cancer *vs*. Normal for 19 kinds of epithelial cancers. We finally obtained 106 unique clusters for these cancer types. A cluster was designated to be homogeneous if at least half of its members show the same direction of expression pattern (either up- or down-regulated). For each cluster, we computed the homogeneous fraction as that of co-dysregulation throughout all cancer types analyzed. A significant *P* value for this fraction was calculated as follow: for each cancer type, the expression of all its associated miRNAs was distributed randomly within these miRNAs for 10,000 times, keeping the distribution of up- and down-regulated miRNAs constant for each step. The homogeneous fraction over all cancers was computed, which yields the *P* value as the number of sampled homogeneous fractions exceeding the original homogeneous fractions divided by 10,000.

In order to check whether clustered miRNAs are more enriched in cancer development compared to single miRNA, we calculated an enrichment score of log-odds (LOD) score for each cancer type:

$$LOD=\log_{2}(\left(\frac{X_{c}}{X_{c}+Y_{c}}\right)/\left(\frac{X_{\mathrm{all}}}{X_{\mathrm{all}}+Y_{\mathrm{all}}}\right))$$

where *X_c_* and *Y_c_* separately denote the numbers of clustered miRNAs and non-clustered miRNAs for each cancer type; *X_all_* denotes the number of the clustered miRNAs, and *Y_all_* denotes the number of the miRNAs not contained in any cluster. Here we tool into account all known human miRNAs annotated in the human genome, thus designating them as the 688 clustered miRNAs and 900 non-clustered miRNAs. In this case, a positive LOD score indicates enrichment for clustered miRNAs compared to non-clustered miRNAs in a specific cancer.

## Implementation and results

### Database content

In the current release of dbDEMC, the data of miRNA transcriptome of total 46,388 samples from 403 studies of human, mouse, or rat were collected from public resources (Table S2). These profiles are derived from 149 subtypes or cell lines from 40 different cancers (Table S3). We then performed a systematic analysis on each dataset, and yielded a total of 807 experiments for differential expression analysis. dbDEMC 3.0 now hosts a total of 3268 DEMs, and among them, 2584 are specific to human. A total of 160,799 miRNA variations related to cancers have been deposited in our database. The detailed information about the numbers of miRNAs, cancer types, datasets, and experiments for different species is presented in Table 1.

**Table 1:** **Summary of the data content of the current release of dbDEMC**

	**No. ofmiRNAs**	**No. of cancer types**	**No. of cancer subtypes**	**No. of datasets**	**No. ofexperiments**	**No. of samples**
*Homo sapiens*	2584	40	149	373	763	45,974
*Mus musculus*	610	11	15	28	40	383
*Rattus norvegicus*	74	2	22	2	4	31
All	3268	40	149	403	807	46,388

**Figure 2:**
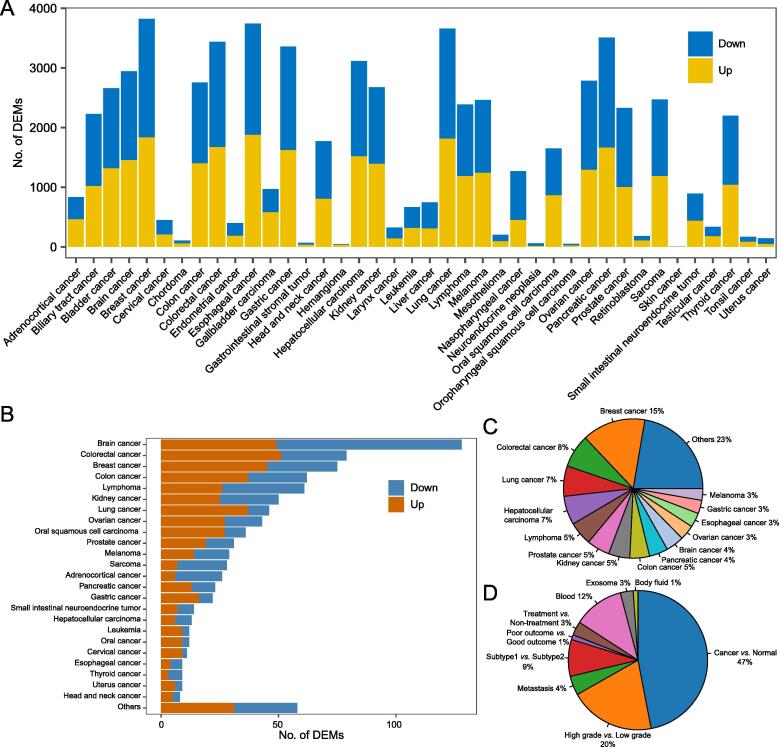
**Statistics of data content in dbDEMC 3.0 for humans** **A.** Number of DEMs from each cancer type identified by high-throughput methods. **B.** Number of DEMs from major cancer types identified by low-throughput methods. **C.** Percentage of experiments for major cancer types. **D.** Percentage of experiments in nine types of experimental design. DEM, differentially expressed miRNA.

Figure 2A depicts the number of DEMs for each type of human cancers. For example, the breast cancer presents a large number of DEMs with 1833 up-regulated and 1988 down-regulated. The number of DEMs from mouse and rat can be found in Figure S1. Figure 2B demonstrates the number of DEMs validated by low-throughput methods across major cancers, and the brain cancer, colorectal cancer, and breast cancer are top ranked cancer types. Figure 2C shows the percentages of experiments for top ranked cancers. The breast cancer accounted 15% of the total experiments, and ranked the first of the list, followed by colorectal cancer and lung cancer. Whereas for the 9 different comparison categories, cancer samples *vs*. normal controls constitutes about half of the total experiments, followed by the comparison of high-grade *vs*. low-grade cancer samples (Figure 2D). Overall, the sizes of analysis experiments and related literatures in dbDEMC 3.0 have a two-fold increment by comparing with the previous version (Figure S2).

### New features

In the dbDEMC 2.0, we assigned the different experimental designs to 7 different categories: Cancer *vs*. Normal, High grade *vs*. Low grade, Metastasis *vs*. Primary cancer, Subtype1 *vs*. Subtype2, Poor outcome *vs*. Good outcome, Blood sample of patients *vs*. Blood sample of normal controls, and also Treatment *vs*. Non-treatment. In recent years, many studies disclosed that exosomes and microvesicles act as cell communication agents, where miRNAs are the most important molecular in exosomes and microvesicles that play a role in regulating cancer progression [29]. In addition, circulating miRNAs have also been widely found in body fluids and represent a gold mine of noninvasive biomarkers in cancer [30]. In this update version, we thus added these two categories of experimental design: Exosome sample from patients *vs*. Exosome sample from control, and Body fluid from patients *vs*. Body fluid from control (Figure 2D). Moreover, for each DEM set, targets of miRNAs and enrichment information of the target genes for the KEGG pathways and GO terms were deposited in the dbDEMC 3.0, which makes it possible for inspecting functional mechanisms behind a set of miRNAs.

### Newly designed web interface

**Figure 3:**
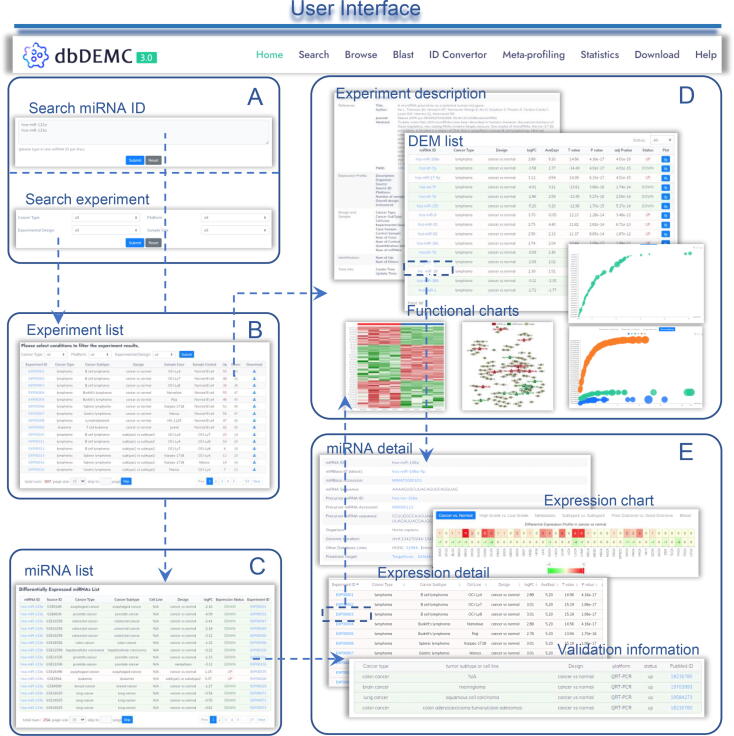
**Web interface of dbDEMC 3.0** **A.** Search page. miRNAs can be searched via miRBase IDs or filtering experiments with interested conditions. **B.** Filtering result page of experiments. **C.** Search result page with example miRNAs. **D.** Experiment page. The page summarizes the description of the experiments and the associated DEM list. The functional chart, including differential expression heatmap, regularly network, and miRNA target-enriched KEGG pathways and GO terms, is also depicted. **E.** miRNA page. This page mainly consists of four sections: miRNA summary, expression profile, expression detail, and validation.

The web interface of dbDEMC 3.0 has been significantly refined and improved, allowing better use of the deposited data. The Search page permits users to perform a quick search and extract summarized information of a DEM list across cancer types. Users can also specify the cancer type, experimental design, or platform to select the interested experiments (Figure 3A–C). After filtering the experimental results, users can select interested experiments. Detailed information of DEM-related experiments, which includes the description with the up-regulated and down-regulated miRNAs, can be accessed. In the functional chart section, heatmap of the differential expression, miRNA–target regulatory network for top ranked DEMs, and the bubble chart for miRNA target-enriched KEGG pathways and GO terms are presented (Figure 3D). Using a single miRNA query, summary information of the interested miRNA can be retrieved, including the general description of the interested miRNA and the differential expression summary heatmap which depicts the number of experiments showing up- or down-regulation. In addition, summary statistics tables for both high-throughput data analysis and low-throughput validation data are also displayed (Figure 3E).

### Analyzing tools

miRBase is the central reference database for miRNA annotation by assigning names and unique gene IDs for each miRNA. During its development, some miRNA definition and annotation may have been changed. This leads to the inconsistence of the miRNA IDs from different datasets, which are derived from different miRBase versions and make it difficult for comparing research results for integrative analysis. To solve this problem, we provide a “ID convertor” in our database, by which users could convert miRBase old version IDs to the latest version (v22.0) for the three species of human, mouse, and rat. In addition, other analyzing tools including BLAST and meta-profiling, which are used for sequence similarity search of unknown miRNAs and identify the confident cancer-related miRNAs in pan-cancer wide, are also available in dbDEMC v3.0. For the meta-profiling study, the vote-counting approach is used to calculate the consistent score of differential expression for meta-analysis [31]. Common miRNAs identified in multiple cancer types with a similar differential expression pattern suggest that they may have similar regulatory mechanisms and play important roles in cancer development.

### miRNA clusters are significantly overrepresented in cancers

A large proportion of miRNAs are localized as conserved clusters in the genome and present a similar expression pattern across tissues. It is critical to understand whether miRNA clusters present a similar differential expression pattern across cancers and correlate with the similar pathobiology. Here we obtained human miRNA cluster annotation from public resources, which includes 22.8% (688/2588) of mature miRNAs appearing as 143 clusters of at least two members within (Table S4). We systematically analyzed the homogeneity of expression patterns within miRNA clusters. We excluded those clusters having less than half of all miRNAs annotated from the results of TCGA, which leads to 106 remaining clusters. The clusters are denoted as exhibiting a homogeneous expression pattern if annotated miRNA members are either up- or down-regulated (see Data processing). In total, cancer-associated clusters revealed homogeneous expression patterns for 74% of all annotated cancers, which confirms the hypothesis of a co-regulation pattern of miRNA clusters in cancer. For example, the cluster of miR-142-5p, miR-142-3p, and miR-4736 presents a consistent differential expression pattern in 91% (11/12) of the cancers analyzed (Table S5). A null model by randomly linking miRNA expression patterns (permutation 10,000 times within each cancer) indicated that 52 clusters (49%, *P* < 0.05) showed a significantly higher homogeneity pattern in all 19 kinds of cancer compared to that expected by chance (Table S5). These clusters exhibit a homogeneous expression pattern in at least 78.5% of all these types of cancer.

**Figure 4:**
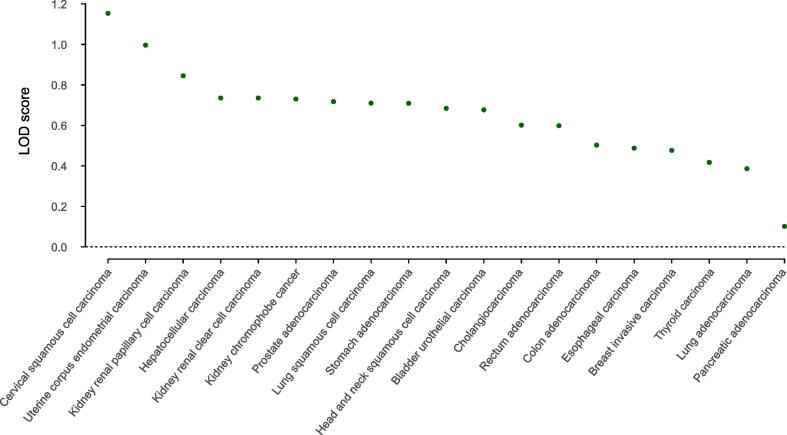
**miRNA clusterenrichment for 19 kinds of cancer** For each cancer type, the LOD score is plotted. There is an enrichment of miRNA cluster members for all 19 kinds of cancer (100%, *P* < 1E−4). Within these 19 types of cancer, miRNAs located in clusters are, on average, 1.56 times (LOD = 0.65) enriched compared to random permutation. LOD, log-odds.

To further investigate the association of miRNA clusters with different kinds of cancer, we estimated the enrichment of miRNA clusters in cancer-associated miRNAs by using a LOD score. We found enrichment for all 19 kinds of cancer (Figure 4). Within these 19 kinds of cancer, miRNAs located in clusters are, on average, 1.56 times (LOD = 0.65) enriched compared to random permutation. In summary, our analyses show a significant enrichment of clustered miRNAs in cancers compare to the single miRNA members, demonstrating that different miRNAs within a cluster act synergistically in cancer development.

## Discussion

Over the last decade, a large number of miRNA transcriptome profiles of various cancers have been generated. Many studies have performed miRNA transcriptome analysis to explore the underlying molecular mechanisms of miRNA genes in cancer development [32], [33]. This progress motivated a novel release of dbDEMC to keep track of the latest published data. Along with this, we curate these data and provide a platform to facilitate the study of miRNA–cancer associations. For dbDEMC 3.0, it not only contains more miRNA–cancer associations, we also extend our database to the species of mouse and rat, which will be of benefit to those studies characterizing the miRNA functional machinery in cancer using the model organisms. Beyond the rapid increase of data amount, our database now offers many new features and powerful tools for the downstream analysis of DEMs, such as the integrated target identification and functional enrichment analysis for miRNA-regulated biological processes.

One of the key questions of differential expression analysis of miRNAs is which cancer types are regulated by a particular miRNA (miRNA-centric view), or conversely, which miRNAs may be involved in a given type of cancer (cancer-centric view). Our database supports both miRNA- and cancer-centric investigations, *i.e.*, users are able to search miRNAs to determine the spectrum of cancer types that are involved in, or to find a candidate miRNA list which links to an individual type of cancer. It is worth noting that previous studies have indicated that false positive and negative records may exist in miRBase, thus researchers need to be cautious about resources based on references from miRBase [34], [35]. In addition, our database hosts miRNAs that present differential expression in cancers by using high-throughput methods, thus most of miRNAs in the human genome are included. Researchers could further explore their roles in cancer development and identify those “*bona fide*” cancer driver miRNAs. Overall, we expect that dbDEMC 3.0 could serve as a valuable resource with comprehensive data amount and data analysis tools to facilitate the study of DEMs in cancers. In the future, more data from other public resources such as International Cancer Genome Consortium (ICGC) [36] and Chinese Glioma Genome Atlas (CGGA) [37], will be added. We will also continue to make improvements to the web interface of our database for the flexible analysis of miRNA functions. We believe that the development of dbDEMC database can help accelerate the integration between miRNANome and cancer studies.

## Data availability

dbDEMC v3.0 is freely accessible at https://www.biosino.org/dbDEMC/.

## CRediT author statement

**Feng Xu:** Data curation, Formal analysis. **Yifan Wang:** Methodology, Validation. **Yunchao Ling:** Visualization. **Chenfen Zhou:** Data curation. **Haizhou Wang:** Data curation. **Andrew E. Teschendorff:** Methodology, Software. **Yi Zhao:** Investigation, Software. **Haitao Zhao:** Validation. **Yungang He:** Supervision, Investigation. **Guoqing Zhang:** Methodology, Supervision. **Zhen Yang:** Conceptualization, Supervision, Writing - original draft. All authors have read and approved the final manuscript.

## Competing interests

The authors have declared no competing interests.

## Supplementary material

Supplementary Figure S1 **Number of DEMs identified by high-throughput methods for each cancer type A.** Number of DEMs for mouse; **B.** Number of DEMs for rat.

Supplementary Figure S2 **Increasing number of experiments for each cancer type** The number of experiments for each cancer type in dbDEMC v3.0 and v2.0 are depicted.
