# Correction to: dbDEMC 3.0: Functional Exploration of Differentially Expressed miRNAs in Cancers of Human and Model Organisms

**DOI:** 10.1093/gpbjnl/qzae037

**Published:** 2024-05-25

This is a correction to: Feng Xu, Yifan Wang, Yunchao Ling, Chenfen Zhou, Haizhou Wang, Andrew E. Teschendorff, Yi Zhao, Haitao Zhao, Yungang He, Guoqing Zhang, Zhen Yang, dbDEMC 3.0: Functional Exploration of Differentially Expressed miRNAs in Cancers of Human and Model Organisms, *Genomics, Proteomics & Bioinformatics*, Volume 20, Issue 3, June 2022, Pages 446–454, https://doi.org/10.1016/j.gpb.2022.04.006.

The published version of this manuscript contained errors in the author affiliation listings. The corrected affiliations are as follows:

Feng Xu^1,#^, Yifan Wang^2,#^, Yunchao Ling^2^, Chenfen Zhou^2^, Haizhou Wang^1^, Andrew E. Teschendorff^3^, Yi Zhao^4^, Haitao Zhao^5^, Yungang He^6,*^, Guoqing Zhang^2,*^, Zhen Yang^1,*^

^1^ Center for Medical Research and Innovation of Pudong Hospital, Fudan University Pudong Medical Center, and Shanghai Key Laboratory of Medical Epigenetics, International Co-laboratory of Medical Epigenetics and Metabolism (Ministry of Science and Technology), Institutes of Biomedical Sciences, Fudan University, Shanghai 200032, China

^2^ Bio-Med Big Data Center, CAS Key Laboratory of Computational Biology, Shanghai Institute of Nutrition and Health, University of Chinese Academy of Sciences, Chinese Academy of Sciences, Shanghai 200031, China

^3^ CAS Key Laboratory of Computational Biology, Shanghai Institute of Nutrition and Health, University of Chinese Academy of Sciences, Chinese Academy of Sciences, Shanghai 200031, China

^4^ Institute of Computing Technology, Chinese Academy of Sciences, Beijing 100190, China

^5^ Department of Liver Surgery, Peking Union Medical College Hospital, Chinese Academy of Medical Sciences and Peking Union Medical College, Beijing 100730, China

^6^ Shanghai Fifth People’s Hospital, and Shanghai Key Laboratory of Medical Epigenetics, International Co-laboratory of Medical Epigenetics and Metabolism (Ministry of Science and Technology), Institutes of Biomedical Sciences, Fudan University, Shanghai 200032, China

These details have been corrected only in this correction notice to preserve the published version of record.

