# Supplementary figures and images for: dbDEMC 3.0: Functional Exploration of Differentially Expressed miRNAs in Cancers of Human and Model Organisms
Source: Genomics Proteomics Bioinformatics. 2022 May 25;20(3):446–54. doi: 10.1016/j.gpb.2022.04.006 (PMC9801039; doi:10.1016/j.gpb.2022.04.006)

Status DOWN UP

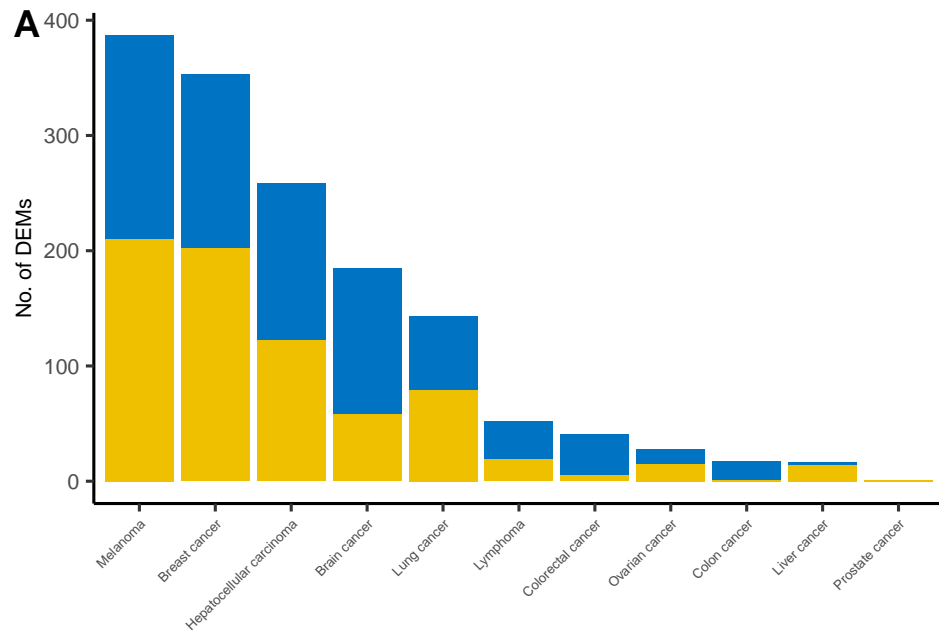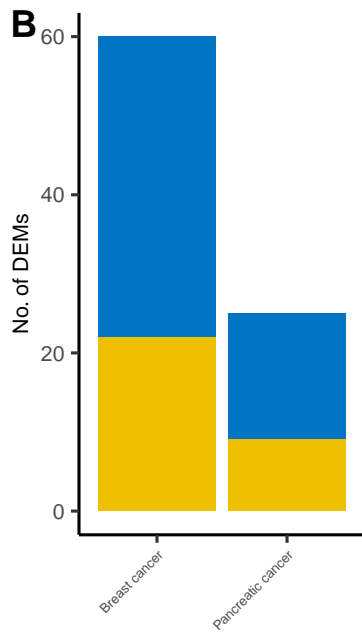

Supplement: Supplementary Figure S1 — Number of DEMs identified by high-throughput methods for each cancer type A. Number of DEMs for mouse; B. Number of DEMs for rat. [file mmc1.pdf]

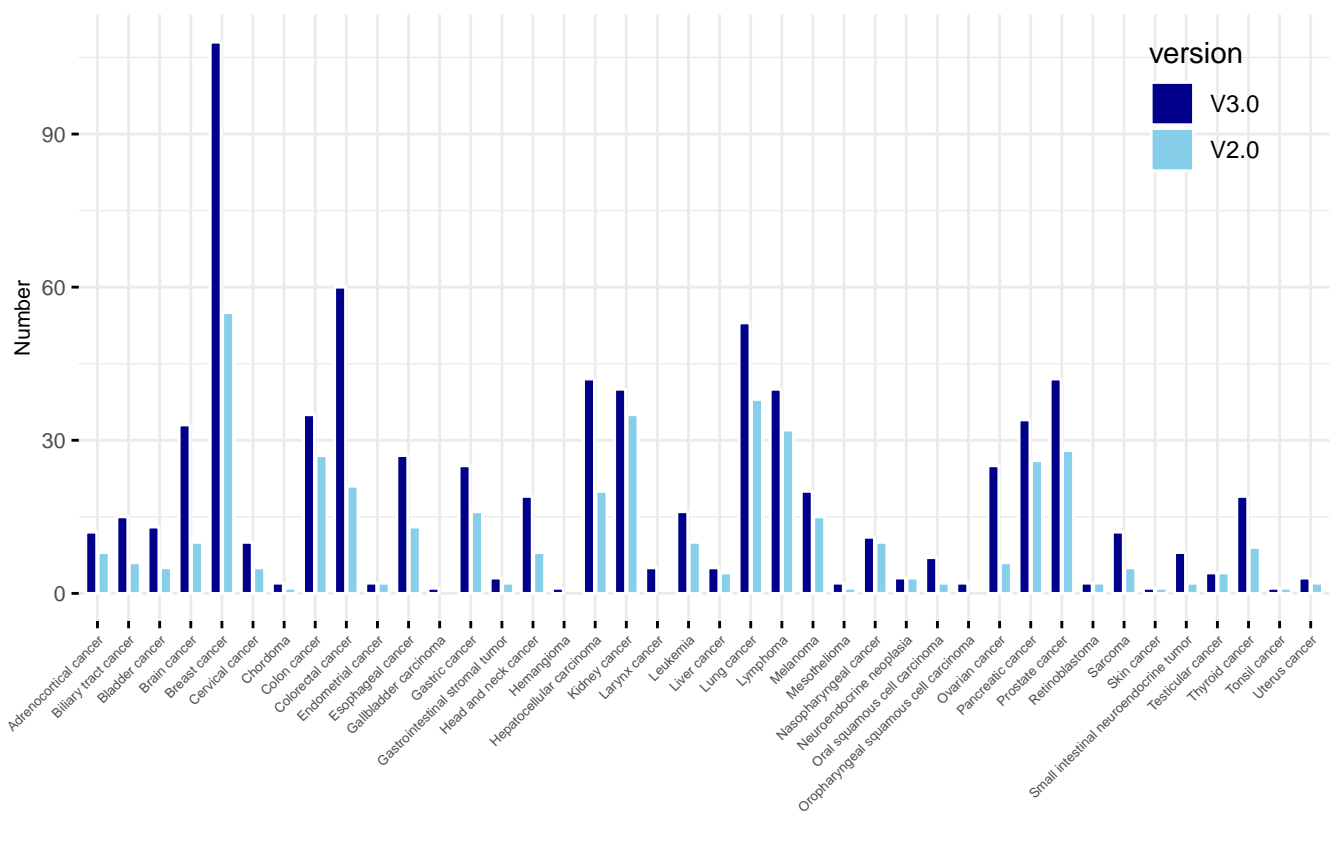

Supplement: Supplementary Figure S2 — Increasing number of experiments for each cancer type The number of experiments for each cancer type in dbDEMC v3.0 and v2.0 are depicted. [file mmc2.pdf]
