# Supplementary material for: dbDEMC 3.0: Functional Exploration of Differentially Expressed miRNAs in Cancers of Human and Model Organisms
Source: Genomics Proteomics Bioinformatics. 2022 May 25;20(3):446–54. doi: 10.1016/j.gpb.2022.04.006 (PMC9801039; doi:10.1016/j.gpb.2022.04.006)
Supplement: Supplementary Table S1 [file mmc3.docx]

**Table S1 Adapters for miRNA-seq kits for the Illumina platform**

| **Kit** | **Vendor** | **3' Adapter sequence** |
| --- | --- | --- |
| TruSeq Small RNA Library Preparation Kit | Illumina | 5'-TGGAATTCTCGGGTGCCAAGG-3' |
| NEXTflex Small RNA Sequencing Kit ** | PerkinElmer | 5'-TGGAATTCTCGGGTGCCAAGG-3' |
| NEBNext Multiplex Small RNA Library Prep Kit for Illumina | New England Biolabs | 5'-AGATCGGAAGAGCACACGTCT-3' |
| TailorMix miRNA Sample Preparation Kit | SeqMatic | 5'-TGGAATTCTCGGGTGCCAAGG-3' |
| CleanTag Small RNA Library Prep Kit | TriLink | 5'-TGGAATTCTCGGGTGCCAAGG-3' |
| QIAseq miRNA Library Kit | Qiagen | 5'-AACTGTAGGCACCATCAAT-3' |
| Small RNA-Seq Library Prep Kit | Lexogen | 5'-TGGAATTCTCGGGTGCCAAGGAACTCCAGTCAC-3' |
| SMARTer smRNA-Seq Kit for Illumina | Takara | 5'-AAAAAAAAAA-3' |
| CATS small RNA-seq Kit | Diagenode | 5'-GATCGGAAGAGCACACGTCTG-3' |
