# Supplementary material for: dbDEMC 3.0: Functional Exploration of Differentially Expressed miRNAs in Cancers of Human and Model Organisms
Source: Genomics Proteomics Bioinformatics. 2022 May 25;20(3):446–54. doi: 10.1016/j.gpb.2022.04.006 (PMC9801039; doi:10.1016/j.gpb.2022.04.006)
Supplement: Supplementary Table S3 [file mmc5.docx]

**Table S3 Cancer types and associated subtypes and cell line names covered in dbDEMC 3.0**

| **Cancer type** | **Cancer subtypes** | **Cell line** |
| --- | --- | --- |
| Adrenocortical cancer | Adrenocortical carcinoma |  |
| Biliary tract cancer | Cholangiocarcinoma, Cholangiocellular carcinoma, Distal cholangiocarcinoma |  |
| Bladder cancer | Bladder urothelial carcinoma |  |
| Brain cancer | Medulloblastoma, Glioblastoma, Meningioma, Schwannoma tumors, Glioma, Anaplastic astrocytoma, Glioblastoma multiforme, Brainstem gliomas, Astrocytoma, Pilocytic astrocytoma |  |
| Breast cancer | ER positive, ER negative, Luminal A, Luminal B, HER2+, Basal-like, Normal-like, PR positive, PR negative, Invasive ductal carcinoma, Triple negative, Invasive lobular carcinoma, Squamous cell breast carcinoma, Acantholytic variant | MCF-7, LCC9, LCC2, 67NR, 168FARN, 4TO7, 4T1 |
| Cervical cancer | Cervical adenocarcinoma, Cervical squamous cell carcinoma |  |
| Chordoma |  |  |
| Colon cancer | Colonic adenomas, Tubulovillous adenoma, Serrated adenoma, Colon adenocarcinoma | SW620, LoVo, FHC |
| Colorectal cancer | Duke A, Duke B, Duke C, Colorectal adenocarcinoma | HCT116, DKO-1, Dks-8, DLD-1 |
| Endometrial cancer | uterine corpus endometrial carcinoma |  |
| Esophageal cancer | Barrett's carcinogenesis, Esophageal squamous cell carcinomas, Esophageal adenocarcinoma | OE19 |
| Gallbladder carcinoma |  |  |
| Gastric cancer | H. Pylori positive gastric cancer, H. Pylori negative gastric cancer |  |
| Gastrointestinal stromal tumor |  |  |
| Head and neck cancer | HPV positive, HPV negative, Head and neck squamous cell carcinoma | Cal 27, H413, HGEPp, Detroit 562, FaDu |
| Hemangioma | Infantile hemangioma |  |
| Hepatocellular carcinoma | Hepatocellular carcinoma, Hepatoblastoma | HepG2 |
| Kidney cancer | Wilms tumor, Clear cell renal cell carcinoma, Papillary renal cell carcinoma, Chromophobe renal cell carcinoma, Oncocytoma, Kidney renal clear cell carcinoma, Kidney chromophobe cancer |  |
| Larynx cancer | Laryngeal squamous cell carcinoma |  |
| Leukemia | T cell leukemia, Acute lymphocytic Leukemia, Acute myelogenous leukemia, B acute lymphoblastic leukemia, T acute lymphoblastic leukemia, Acute myelocytic leukemia, Chronic lymphocytic leukemia, Chronic myeloid leukemia, Plasma cell leukemia | Jurkat |
| Liver cancer | intrahepatic cholangiocarcinoma, Combined hepatocellular Cholangiocarcinoma |  |
| Lung cancer | Lung squamous cell carcinoma, Lung adenocarcinoma, Small cell lung cancer, Large cell neuroendocrine cancer, Large cell carcinoma, Non-small cell lung cancer | H446, LLC1 |
| Lymphoma | B cell lymphoma, Burkitt's lymphoma, Splenic lymphoma, Gastric lymphoma, Lymphoblastoid, Follicular cleaved Lymphoma, Diffuse large B cell lymphoma, Multiple myeloma, B cell lymphoma of MALT type, B-cell non-Hodgkin lymphoma, Hodgkin lymphoma, myeloma, T-cell lymphoblastic lymphoma, Primary central nervous system lymphomas, Diffuse large B-cell lymphoma, MYC-dependent lymphoma, CD4+ T cell lymphomas, Chronic myelomonocytic leukemia | OCI-Ly4, OCI-Ly7, OCI-Ly8, Namalwa, Raji, Karpas-1718, Manca, HG-1125, H929 |
| Melanoma | Skin cutaneous melanoma, Melanoma brain metastasis | A375, A375R, M117,M113, B16F0 |
| Mesothelioma | Malignant pleural mesothelioma, Diffuse malignant peritoneal mesothelioma |  |
| Nasopharyngeal cancer |  |  |
| Neuroendocrine neoplasia |  |  |
| Oral squamous cell carcinoma |  |  |
| Oropharyngeal squamous cell carcinoma |  |  |
| Ovarian cancer | Clear cell ovarian carcinoma, High-grade serous ovarian carcinoma, Familial ovarian cancer, High grade serous papillary ovarian cancer, Serous ovarian cancers | HIO180, SKOV3_ip1, SKOV3_TR, HEYA8, A2780 |
| Pancreatic cancer | Pancreatic ductal adenocarcinoma, Solid-pseudopapillary neoplasm of pancreas, Pancreatic adenocarcinoma | PaCa-2, S2-013, PANC-1, ASML |
| Prostate cancer | Prostate adenocarcinoma, Castration-resistant prostate cancer |  |
| Retinoblastoma |  |  |
| Sarcoma | Synovial sarcoma, Liposarcoma, Osteosarcoma, Kaposi's sarcoma, Rhabdomyosarcomas, Synovial sarcoma |  |
| Skin cancer |  |  |
| Small intestinal neuroendocrine tumor |  |  |
| Testicular cancer | Testicular germ cell tumor |  |
| Thyroid cancer | Primary papillary thyroid carcinomas, Medullary thyroid cancer, Papillary thyroid carcinoma, Follicular thyroid carcinoma |  |
| Tonsil cancer |  |  |
| Uterus cancer |  |  |
